# Delivering the Parenting for Lifelong Health Programme with Parents of Young Children in Wales

**DOI:** 10.3390/children12101280

**Published:** 2025-09-23

**Authors:** Judy Hutchings, Sarah Jones, Anwen Jones, Margiad Williams, Jamie Lachman

**Affiliations:** 1 School of Psychology, Brigantia Building, Bangor University, Bangor LL57 2AS, UK; anwen.r.jones@bangor.ac.uk; 2 Barnardo’s, Caban Bach, Meirion Terrace, Blaenau Ffestiniog LL41 3UA, UK; sarahjanejones@gwynedd.llyw.cymru; 3 School of Educational Sciences, Bangor University, Bangor LL57 2PZ, UK; margiad.williams@bangor.ac.uk; 4 Department of Social Policy and Intervention, University of Oxford, Barnett House, 32-37 Wellington Square, Oxford OX1 2ER, UK; jamie.lachman@spi.ox.ac.uk

**Keywords:** parenting challenges, children, social disadvantage, parenting intervention, UK

## Abstract

**Highlights:**

**What are the main findings?**

- Child behaviour challenges and parenting challenges have risen since COVID-19, especially for low-income families.
- The cost of delivering parenting interventions can be substantial.

**What is the implication of the main finding?**

- The Parenting for Lifelong Health programme for parents of Young Children (PLH-YC) is an evidence-based low-cost intervention.
- Results suggest that the programme is feasible, acceptable, and achieves good outcomes with vulnerable families in Wales.

**Abstract:**

Background/Objectives: Based on years of work from high-income countries, the Parenting for Lifelong Health programme for parents of Young Children (PLH-YC) was developed by the first and last authors, as a freely available low-cost programme for low-income families in low- and middle-income countries (LMICs). The initial group-based 12-session programme has since been delivered, adapted, and evaluated across many LMICs and now has a significant body of evidence. Over the last 10 years, early intervention services in the UK have been considerably reduced whilst, exacerbated by the impact of COVID-19, service demands have grown. This paper describes a feasibility trial of the 12-session PLH-YC programme in Wales to explore whether it could recruit and retain parents, and demonstrate improvements in parenting skills and reductions in child behaviour problems. Methods: Two small pre–post trials were conducted in socially disadvantaged communities in Wales, and they were delivered by local parenting practitioners. Of the 20 parents recruited across 3 groups, 17 provided pre- and post-course data and 10 completed qualitative interviews. Results: Retention was good (85%) with mean attendance of 8.7 sessions, and parental and facilitator feedback reported high levels of satisfaction with the programme, with the only recommendation being to make the programme longer and for facilitators to be given more time. Results showed significant benefits to parent-reported parenting practices, child behaviour, and parental mental wellbeing. Conclusions: These preliminary results justify work to develop a rigorous evaluation to establish whether PLH-YC could have a place among parenting-support programmes in the UK.

## 1. Introduction

There is strong evidence of a connection between coercive and inconsistent parenting and childhood behavioural difficulties that predicts a range of ongoing difficulties into adulthood [1,2,3,4]. The global prevalence of violence against children is estimated at over half of all children experiencing violence within the previous year, amounting to over 1 billion of the world’s children [5]. Consequently, many parenting interventions focus on teaching parents to establish positive and non-aversive parenting skills [6], with the strongest evidence coming from programmes based on social learning theory [6,7]. These programmes demonstrate significant increases in positive parenting behaviours, improvements in parental mental health, and in promoting children’s development and reducing challenging behaviour and child behavioural problems [7,8].

The well-established programmes with the best evidence initially focus on building positive relationships with children through child-led activities, then creating a consistent and predictable environment to increase positive behaviour, and, finally, on introducing planned non-aggressive management of problem behaviour. This approach has continued to be the basis of most of the effective programmes [8,9].

### 1.1. Social Disadvantage

Both longitudinal [1] and intervention studies [6,10] from high-income countries (HICs) have repeatedly shown that the risks for children from poor parenting are greater when families live in conditions of socioeconomic adversity [11], something that occurs at considerably higher rates in LMICs [5]. However, trials of programmes developed in high-income countries (HICs) have shown that they are both transportable and effective in such settings [12], demonstrating that the core social learning theory principles [3,13,14,15] are globally relevant. Unfortunately, many programmes developed for HICs are licenced and too costly for low-income countries that have a much smaller level of spending for healthcare [16]. As a result, low-cost programmes have been developed for LMICs and are demonstrating evidence of their effectiveness. The Parenting for Lifelong Health suite of programmes are an example [17,18].

### 1.2. Parenting for Lifelong Health

The first and last authors were founding members of the initiative that developed the Parenting for Lifelong Health (PLH) suite of programmes. The goal of PLH is to develop and disseminate low-cost, open-source, culturally relevant, parent-centred, and evidence-based programmes [19]. PLH programmes are licenced under a Creative Commons Attribution-Share-alike 4.0 licence. Studies adapting PLH have suggested the importance of context and cultural adaptation on a surface level, in terms of images, language, etc., but that key parenting principles associated with positive child development are consistent across countries [20,21].

### 1.3. Parenting for Lifelong Health Programme for Parents of Young Children (PLH-YC)

The first and last authors initially co-developed the group-based 12-session Parenting for Lifelong Health programme for parents of young (2–9-year-old) children (PLH-YC), which was adapted for isiXhosa-speaking parents. They trained the first cohort of facilitators and contributed to the initial pilot and RCT trials in South Africa [16,19]. They have since supported trials in the Philippines, Thailand, and across Eastern Europe, where the programme has been delivered in different lengths and formats, including online and blended delivery, and good evidence has resulted from a number of trials [16,21,22].

### 1.4. The Situation in Wales

The Sure Start initiative was launched across the UK in 1999 to provide services to families living in relatively disadvantaged communities with children under age five. Longer term benefits from 10 years of Sure Start are now showing positive effects on a range of health [23] and academic outcomes from age 7 lasting through to GCSEs, with the strongest improvements in language and communication skills [24]. The situation in the UK has since changed significantly with funding cuts in early-years support services for families halved from over GBP 3.8 bn to GBP 1.9 bn in a decade [25] and with current spending on these services now at a quarter of what was allocated to Sure Start in 2009/10 [26].

The impact of COVID-19 saw an increase in levels of behavioural challenges among pre-school children in the UK [27,28], and the reductions in local authority funding suggested that, as a low-cost programme delivered by parenting workers, the PLH-YC programme might be a useful addition to parenting support in the UK. This paper describes a feasibility study of PLH-YC in Wales from two small trials exploring its acceptability and preliminary outcomes. The programme was initially delivered in a socially disadvantaged housing estate in North Wales by a community worker. Subsequent interest from another community project in a very socially disadvantaged small ex-slate mining community in North Wales enabled the programme to also be delivered there.

## 2. Methods

### 2.1. Aims

The aim of the projects was to explore the feasibility of recruiting, delivering, and retaining participants in the PLH-YC programme developed for low-income countries, to gather feedback from programme participants and facilitators, and to explore preliminary outcomes for participants living in low-income communities in Wales.

### 2.2. Recruitment

#### 2.2.1. Project One

Recruitment for the initial project was undertaken by a local community worker. The setting was a residential estate, on the outskirts of a small city in North Wales, that has a high level of social housing and social disadvantage with free school meal rates of 30%. The group was delivered in a community centre that offers childcare and family support. Seven parents were recruited from the estate.

#### 2.2.2. Project Two

Recruitment for the second project was undertaken by community workers in an area of North Wales served by a Barnardo’s funded community support programme for children and families. The area had similar levels of social disadvantage to Project One. The community workers were based in a centre that has been delivering parenting support for many years. Two groups of parents were recruited, one with six parents and one with five parents.

### 2.3. Data Collection

In both settings, baseline data was collected by the community workers who undertook recruitment. Follow-up measures were collected by independent researchers based at Bangor University. In project 2, the researcher also undertook recorded follow-up interviews with participants and programme facilitators. Ethical approval was granted by the Bangor University School of Psychology Ethics committee (17259-2023), approved on 2 July 2012.

### 2.4. Intervention

In both projects, the European version of the twelve-session PLH-YC programme, known to parents as the Caring Families programme, was delivered. The programme manual is available on the WHO website [19] and in over 20 languages on the PLH charity website [29].

To keep it low-cost and easy to deliver, the PLH-YC programme uses illustrated stories (comic strips) showing examples of parent and child behaviours that can be easily adapted to different settings. The programme uses the metaphor of building a “House of Support,” to demonstrate the two-stage model [30] in which the walls representing skills to promote positive child behaviour, and the roof represents limit-setting and discipline strategies. Twelve weekly sessions focus on spending quality time with children, naming feelings and actions, using praise and rewards, giving instructions, establishing household rules, redirecting negative behaviours, ignoring attention-seeking and demanding behaviours, using realistic and appropriate consequences, and problem-solving. The programme also includes brief mindfulness-based exercises to support parental mental health and reduce parental stress. The final session reviews what parents have learned, focuses on how to continue applying learned skills at home, and ends with a certificate and celebration. Facilitators make mid-week phone calls to parents to monitor their use of skills at home to encourage programme engagement. In Project One, a community worker (based in the community centre) delivered the programme with the support of the first author. In Project Two, the two groups were both delivered by an experienced parent group programme leader, each with a community worker from the same centre.

### 2.5. Measures

#### 2.5.1. Attendance and Satisfaction

Attendance data was collected by group facilitators. Satisfaction in Project One was measured by a weekly questionnaire administered by the programme facilitators. This asked for a rating of the overall session, group discussion, illustrated stories, and the leaders on a 4-point scale of not helpful, neutral, helpful, or very helpful.

Satisfaction and feedback for Project Two was assessed via semi-structured interviews with parents and practitioners that were collected, recorded, and transcribed by the independent researcher after the final session. Interviews asked parents for their opinions on beneficial elements of the programme, challenges, accessibility, and impact on their parenting behaviour. The facilitators were asked about their background experience and for feedback on delivering the programme.

#### 2.5.2. Outcome Data

All of the measures were drawn from the ones used in other PLH-YC trials

#### 2.5.3. Parenting Skills

These were assessed with the Arnold, O’Leary et al. [31] parenting scale in both projects. This is a 30-item measure that assesses dysfunctional parenting practices. In addition to a total parenting score, there are three subscales: laxness or failure to set and stick to limits, over-reactivity or having arguments with children, and verbosity or threatening consequences that the parent then fails to stick to. Items are scored on a 7-point scale. Higher scores indicate greater dysfunctional parenting. Cut-off scores based on data from the parents of clinic-referred children with behavioural problems are reported [31].

#### 2.5.4. Child Behavioural Challenges

The Strengths and Difficulties Questionnaire [32] was used to assess child behaviour in both projects. This 25-item screening tool assesses child behaviour across four problem subscales (conduct, emotion, hyperactivity, and peer problems) and one prosocial behaviour scale. Items are rated on a 3-point scale of not true, somewhat true, and very true. The total of the four problem subscales gives a total difficulties score. Higher scores indicate more behavioural problems. There is also a prosocial subscale where higher scores represent more positive behaviours.

#### 2.5.5. Parental Mental Health

In Project One, the General Health Questionnaire [33], a 30-item mental health screening tool, was used to assess parental mental health. Items are rated on a 4-point scale from 0 (not at all) to 3 (very much). In Project Two, the Parenting Stress Index-Short Form [34] was used to assess parental mental health. This 36-item measure assesses stress experienced by parents of children up to the age of 12 years. Each item is rated on a 5-point scale from 1 (strongly disagree) to 5 (strongly agree), with higher scores indicating more stress.

### 2.6. Analysis

Interview transcripts from participants and facilitators were read and re-read to generate a list of ideas to enable themes to be identified. Thematic analysis [35] was used to identify common themes across the interviews.

Given the small numbers and the common measures, the Arnold–O’Leary and the SDQ were combined to allow a small but more meaningful assessment of programme acceptability and usefulness. Quantitative analyses were performed to compare baseline and follow-up data using either paired *t*-tests or the non-parametric equivalent (Wilcoxon signed rank test). Effect sizes were calculated, including Cohen’s d for the paired *t*-test and r for the Wilcoxon signed rank test. Cohen’s classifications were used for interpretation (0.1 small, 0.3 medium, and 0.5 large).

## 3. Results

### 3.1. Sample

Of the 20 parents attending any part of the programme, 17 (85%) provided pre- and post-course measures. In Project One, of the seven parents recruited, one withdrew after attending two sessions. Of the remaining six, five parents completed the programme (attended at least eight sessions) and all six provided pre- and post-course data. In Project Two, of the fifteen parents recruited, two withdrew after baseline data collection (before attending any sessions), and two were lost to follow-up with eleven completing pre- and post-course quantitative measures and ten completing the post-course interview.

### 3.2. Demographics

All participating parents were mothers. Just over half of the children were female (52.9%), and the mean child age was just under five years (M = 59.65 months, SD = 33.15).

Baseline characteristics are displayed in Table 1. At baseline, the total and all three parenting sub-scales, laxness, over-reactivity and verbosity scores on the Arnold–O’Leary parenting scale were above the mean for parents of children with behavioural problems, with particularly high scores on the verbosity subscale. For child behaviour, median SDQ scores were within the clinical range for the conduct problems and hyperactivity sub-scales as well as for the total difficulties score. In Project One, parents were not reporting significant levels of mental health problems on the GHQ; however, Project Two parents scored above the clinical cut-off for levels of stress on the PSI.

### 3.3. Attrition and Attendance

Mean attendance for Project One was 7.3 sessions and for Project Two was 9.5 sessions, giving an overall mean attendance for the 17 parents of 8.7 sessions (attendance at 8+ sessions is considered to be an effective intervention exposure [6]. Ten Project Two parents and the three facilitators completed post-course interviews.

### 3.4. Satisfaction

Project One collected satisfaction ratings after each session, with 97% of the overall sessions rated as very helpful and 3% helpful. Very helpful ratings of 89% were reported for group discussion, 92% for illustrated stories, and 97% for leaders, and with no ratings for any category as either neutral or not helpful.

Ten of the eleven parents who provided follow-up data in Project Two completed a post-course satisfaction interview. All ten respondents reported very positive responses to the overall programme, and all aspects of the programme content were referred to, with redirecting and one-on-one time both mentioned by six of the ten respondents. In terms of teaching strategies, problem solving and tips arising from group discussion were mentioned by seven parents, with illustrated stories and group practice both mentioned by six parents. Seven parents reported no challenges with the programme and three reported only logistical challenges, e.g., timing and home practice.

All parents reported positively on the impact on their parenting, with seven stating that they were more emotionally aware, e.g., redirecting behaviours and thoughts, letting things go, apologising, and explaining emotions. Six parents felt that the programme needed more sessions and three that it needed longer sessions. Five parents commented positively on being able to socialise with other parents, four on location and timing being good, and two that they wanted to join more groups in the future.

The three Project Two facilitators completed post-course interviews. One was very experienced in working with families and two were relatively new to the service. All three reported the programme as an enjoyable experience and relevant to their population, with two commenting on the positive relationships and the sharing of ideas between the parents and one on the simplicity of the programme and the quality of the discussion arising from the illustrated stories. The only suggested changes were more time to deliver it and, like the parents, a longer course.

### 3.5. Pre–Post Outcome Measures

Results for the pre- and post-course measures are displayed in Table 2. There were significant reductions in dysfunctional parenting practices with very large effect size benefits for total scores on the Parenting Scale (*d* = 0.71), as well as for the over-reactivity (*d* = 0.87) and verbosity (*d* = 0.68) sub-scales. All scores had reduced to below the clinical cut-offs at follow-up. For child behaviour, there were no significant differences for the total SDQ score or any of the SDQ sub-scales. Conduct problems showed a medium, albeit non-significant, reduction and median scores had moved from the clinical to borderline range at post-course. In terms of parental mental health, there was no significant change for parents in Project One (GHQ). For parents in Project Two, who had a mean baseline score in the clinical range, there was a significant reduction in stress related to parenting a difficult child, with a very large effect size (*d* = 0.79). A non-significant reduction in total stress was also found, again with a large effect size (*d* = 0.57).

## 4. Discussion

Providing parent and caregiver support is key to ensuring positive futures for children at risk of poor long-term outcomes. This is recognised by both the UNICEF [36] and WHO [37] strategies, for establishing positive parenting and protecting children from emotional and physical violence and neglect, that are essential for the achievement of several United Nations Sustainable Development Goals (e.g., Goal 3: Ensuring healthy lives and promoting wellbeing for all at all ages; Goal 4: promoting early childhood development, inclusive and equitable education and lifelong learning; Goal 5: empowering women and girls; and Goal 10: Reducing inequalities within and among countries).

There is, currently, a service delivery crisis in relation to early family support in the UK. Many Sure Start early intervention services set up in the 2000s now barely exist due to financial cuts [24,38]. At the same time, demand for services to support children at risk of poor outcomes has escalated since the COVID-19 pandemic. This suggested that it would be worthwhile undertaking a feasibility trial of the PLH-YC programme in the UK, in this case in Wales, using existing contacts with services that had previously contributed to parenting studies.

The programme was developed as a low-cost, freely available, programme to address the parenting challenges of disadvantaged parents that are associated with poor child outcomes in LMICS where rates of violence against children are high [5]. It has significant evidence from randomised trials in several countries including South Africa, Thailand, the Philippines, and across Eastern Europe [16,21,22]. Its evidence-informed content drew on years of research in high-income countries using core, well established, social learning theory components that inform positive parenting. Early formative studies showed that it required only surface adaptation in terms of language, etc. [21,22]. It was designed to be easily adapted with simple cartoon stories to prompt collaborative discussion about parenting behaviour.

This was the first delivery of the programme in the UK and was a small unfunded Welsh feasibility pilot of the European version of the programme, initially adapted for Montenegro [39]. Within the short time constraints of a Masters project, it was possible to recruit two services to deliver the programme. Parents were recruited through the two centres, both of which provide family support in low-income, socially disadvantaged communities.

Quantitative measures that had previously been used to evaluate similar programmes were selected [6,16]. Baseline data showed clinical levels of problematic parenting behaviour for parents of children with behavioural challenges, and the children scored within the clinical range for behaviour and hyperactivity problems as well as for their total problem scores. For project 2, total parental stress and stress in relation to difficulties associated with their child were in the clinical range. Taken together, this suggests that services had identified and recruited parents with several risk factors for poor child outcomes

Mean attendance over the 12 sessions was high at 8.7 sessions, representing more than two thirds of the programme, which is considered a meaningful dose [6] and aligns with other PLH studies. The trial achieved positive outcomes in terms of retention of participants and positive responses from the satisfaction questionnaires and the qualitative feedback from parents and facilitators.

Quantitative outcomes showed significant reductions in dysfunctional parenting practices, with very large effect-size benefits for total scores on the Parenting Scale (*d* = 0.71) as well as for the over-reactivity and verbosity sub-scales. For child behavioural difficulties, although not significant, the median score reduced from the clinical to the borderline range. In terms of parental mental health problems, there was a significant reduction in stress related to parenting a difficult child with a very large effect.

These results, albeit from a very small sample, suggest that the programme was delivered to a sample of parents in need of support, with children at risk of poor outcomes, and that the programme was showing similar benefits to other PLH-YC programmes.

## 5. Limitations

Lack of selection criteria, the small opportunistic sample with no control group, and dependence on parent-report data undermines the internal validity, the results due to low statistical power, and the lack of opportunity to control for any confounding variables. However, the sample had characteristics that suggested that the parents were experiencing parenting challenges and reported positively on the outcomes.

A limited range of validated measures were used, and, should funding be obtained to undertake a larger, more rigorous RCT trial, the methodology would need considerable tightening, including clarifying recruitment methods and a review of measures. The field of parenting has seen the development of more specific and detailed measures of parenting, such as the Multidimensional Assessment of Parenting Scale (MAPS) developed by Parent and Forehand [40]. There is also some evidence that parents with significant mental health problems do less well in parenting programmes [41], and it was a limitation that the two settings had different measures of parental mental wellbeing. A future study would need to review the current evidence to decide both whom to target and what measures to use to provide a reliable outcome assessment that addresses individual profiles and goals [41].

Whilst programme content is standardised, there are components within the programme that facilitate individualising the way that parents apply it by focusing on parental goals and teaching behavioural principles rather than techniques. Our earlier work has explored the association between parental mental health problems and child behaviour problems and suggests an explanation for how such programmes can be effective at reducing both child behaviour problems and parental depression [42].

## 6. Conclusions

Whilst it is hoped that funding for early intervention services to support children and families will be once again prioritised in the UK, the focus on value for money will remain, and the PLH-YC programme is a low-cost intervention.

The trial brought back to the UK a programme that was developed initially from evidence from high-income countries and then made freely available and delivered in LMICs, where it can potentially reach a wide audience. The results can benefit the literature as they provide a description of the programme to the whole world [19], and its evidence can support researchers in creating new programmes in their own countries.

The purpose of this feasibility trial was to establish whether use of the programme in the UK was feasible and justifies seeking further funds to undertake a more rigorous trial given the growing demand and financial constraints on services.

Taken together, the results, albeit tentatively, show that the programme is feasible, acceptable, meets the needs of vulnerable families, and produces significant changes in their behaviour. This suggests that the PLH-YC programme could be a useful addition to parenting-support services within the UK. The next step would be to explore funding and service interest for a larger rigorous trial.

## Figures and Tables

**Table 1:** Baseline characteristics for parenting behaviour, child behaviour, and parental mental health.

Measures	N	Mean (SD)	Cut-Offs
Parenting skills			
PS Total	17	3.39 (0.65)	3.1
PS Laxness	17	3.15 (1.00)	2.8
PS Over-reactivity	17	3.11 (0.92)	3.0
PS Verbosity	17	3.87 (0.56)	3.4
Child behaviour			
SDQ Total Difficulties	17	17.71 (7.08)	Borderline: 14–16 Clinical: 17–40
		Median (range)	Cut-off
SDQ Emotion symptoms	17	2.00 (0–8)	Borderline: 4 Clinical: 5–10
SDQ Conduct problems	17	4.00 (2–8)	Borderline: 3 Clinical: 4–10
SDQ Hyperactivity	17	9.00 (3–10)	Borderline: 6 Clinical: 7–10
SDQ Peer problems	17	2.00 (1–8)	Borderline: 3 Clinical: 4–10
SDQ Prosocial behaviour	17	7.00 (3–10)	Borderline: 5 Clinical: 0–4
Parental mental health			
General Health Questionnaire (Project 1 only)	6	2.00 (0–3)	5
		Mean (SD)	Cut-off
Parenting Stress Index-Short Form Total score (Project 2 only)	11	103.91 (21.01)	90

**Table 2:** Results of paired *t*-tests and Wilcoxon signed rank test.

Measure	N	Pre Mean (SD)	Post Mean (SD)	*p*	*d* (95% CI)
Parenting skills					
PS Total	17	3.39 (0.65)	2.77 (0.72)	0.010 *	0.71 (0.17, 1.24)
PS Laxness	17	3.15 (1.00)	2.75 (0.94)	0.153	0.36 (−0.13, 0.85)
PS Over-reactivity	17	3.11 (0.92)	2.29 (0.77)	0.002 *	0.87 (0.30, 1.42)
PS Verbosity	17	3.87 (0.56)	3.06 (1.04)	0.013 *	0.68 (0.14, 1.20)
Child behaviour					
SDQ Total	17	17.71 (7.08)	17.12 (7.51)	0.748	0.08 (−0.40, 0.56)
		Median (Range)	Median (Range)	*p*	*r*
SDQ Emotion	17	2.00 (0–8)	3.00 (0–8)	0.805	0.06
SDQ Conduct	17	4.00 (2–8)	3.00 (1–7)	0.099	0.40
SDQ Hyperactivity	17	9.00 (3–10)	8.00 (1–10)	0.361	0.22
SDQ Peer problems	17	2.00 (1–8)	3.00 (0–9)	0.563	0.14
SDQ Prosocial	17	7.00 (3–10)	7.00 (3–10)	0.771	0.07
Parental mental health					
GHQ (project 1 only)	6	2.00 (0–3)	0.50 (0–5)	0.581	0.23
		Mean (SD)	Mean (SD)	*p*	*d* (95% CI)
PSI-SF Total (project 2 only)	11	103.91 (21.01)	89.59 (19.90)	0.086	0.57 (−0.08, 1.20)
PSI-SF Parental Distress	11	35.91 (7.08)	34.00 (8.27)	0.536	0.19 (−0.41, 0.79)
PSI-SF Parent–Child Dysfunctional Interaction	11	28.09 (8.22)	24.09 (7.34)	0.138	0.18 (−0.38, 0.73)
PSI-SF Difficult Child	11	39.91 (9.64)	31.50 (7.29)	0.026 *	0.79 (0.09, 1.45)

* *p* < 0.05.

## Data Availability

All data generated for this study are available from the corresponding author upon reasonable request. The data are not publicly available due to ethical restrictions.

## Abbreviations

The following abbreviations are used in this manuscript:

PLH	Parenting for Lifelong Health
PLH-YC	Parenting for Lifelong Health for parents of Young Children
LMICs	Low- and middle-income countries
HICs	High-income countries
GCSEs	General Certificate of Secondary Education
SDQ	Strengths and Difficulties Questionnaire
GHQ	General Health Questionnaire
PSI	Parenting Stress Index
PSI-SF	Parenting Stress Index-Short Form
